# Feasibility, Efficiency, and Safety of Zero-Fluoroscopy Catheter Interventions for Right-Sided Cardiac Arrhythmias Using Only Electroanatomic Mapping

**DOI:** 10.1159/000526564

**Published:** 2022-08-17

**Authors:** Daniel Hofer, Jan Steffel, Firat Duru, Vera Graup, Tom Sasse, Ardan Saguner, Alexander Breitenstein

**Affiliations:** ^a^Division of Electrophysiology, Department of Cardiology, University Heart Center, University Hospital Zurich, Zurich, Switzerland; ^b^Division of General Internal Medicine, Triemli Hospital, Zurich, Switzerland

**Keywords:** Zero-fluoro, Electroanatomic mapping, Fluoroscopy, Ablation, Electrophysiology

## Abstract

**Introduction:**

Fluoroscopy is traditionally used for catheter interventions in electrophysiology but carries a long-term health risk. Besides additional invasive procedures to achieve zero-fluoroscopy (ZF) interventions, electroanatomic mapping may be an alternative to fluoroscopy without the need of additional procedures. We aimed to investigate the feasibility, safety, and efficiency of a ZF approach using only electroanatomic mapping (ZF) compared to a conventional fluoroscopic (CF) approach for patients with right sided cardiac arrhythmias.

**Methods:**

We performed a single centre retrospective cohort study of consecutive patients undergoing catheter interventions for electrophysiologic procedures from January 2019 to December 2020. Patients with left-sided arrhythmias, focal cryoablation, implanted endocardial devices, or additional interventions requiring fluoroscopy were excluded.

**Results:**

202 patients underwent a ZF and 126 patients underwent a CF approach for right-sided cardiac arrhythmias. Apart from atrial fibrillation (ZF 16% vs. CF 9%, *p* = 0.044), baseline demographics were similar in both groups. Acute success rate was 100% in the ZF group and 97.9% in the CF group. Mean procedure time was lower in the ZF group (70 ± 36 vs. 87 ± 44 min, *p* = 0.0001), while ablation time (356 ± 324 vs. 320 ± 294 s, *p* = 0.157) was similar. Total complication rate was low in general (1.0 % major, 2% minor complications) and without a difference between both groups.

**Conclusion:**

A ZF approach using only electroanatomic mapping without additional invasive procedures to diagnose and treat right-sided cardiac arrhythmias is feasible, efficient, and safe.

## Introduction

To navigate catheters during catheter interventions for electrophysiological (EP) procedures, fluoroscopy and three-dimensional navigations systems with electroanatomical mapping capabilities can be used. Fluoroscopy during catheter interventions for right-sided cardiac arrhythmias has historically been estimated with an effective radiation dose of 3–4 mSv per procedure, an exposure 350–2,000 times higher than a chest X-ray, leaving background radiation for more than a year [1]. Accordingly, physicians and nurses working in EP laboratories represent the majority of medical staff receiving the highest annual radiation exposure, leading to an estimated lifetime attributable cancer risk of 1 out of 200, increased incidence of left-sided brain cancer and radiation-induced subcapsular cataracts, and orthopaedic problems because of routine wearing of protective lead aprons [2, 3].

The use of advanced electroanatomic mapping systems has increased over the last years, leading to reduced fluoroscopy exposure for patients and staff. Because of deterministic and stochastic effects, the residual health risk is difficult to determine, but even low amounts of radiation exposure may result in an increased long-term risk of cancer [4]. Current recommendations favour even complete non-ionizing approaches if comparable safety and efficacy is warranted [4]. Previous attempts at eliminating fluoroscopy have mainly been performed with additional pre- or periprocedural invasive procedures such as intracardiac echocardiography (ICE). The aim of this study was to evaluate the feasibility, safety, and efficiency of a zero-fluoroscopy (ZF) approach using only electroanatomic mapping for diagnosis and treatment of right-sided cardiac arrhythmias.

## Methods

### Study Design

Between January 2019 and December 2020, consecutive patients undergoing catheter interventions for diagnostic electrophysiological study (EPS), catheter ablation of supraventricular tachycardia (cavotricuspid isthmus [CTI]-dependent atrial flutter, right-sided atrial tachycardia [AT], atrioventricular-nodal reentry-tachycardia [AVNRT], or Wolff-Parkinson-White [WPW] using a right-sided accessory pathway) or premature ventricular contraction with right ventricular origin were included into this retrospective single centre cohort study. For simplification purposes, concealed accessory pathways with retrograde atrioventricular reentry-tachycardias were subsumed in the WPW group. Since we perform trans-septal puncture under fluoroscopic guidance only, patients with arrhythmias of left-sided origin were excluded. Similarly, patients with implanted transvenous devices or undergoing focal cryoablation necessitating fluoroscopy were excluded. Patients with additional procedures during the same intervention necessitating fluoroscopy (i.e., closure of a persistent foramen ovale) were also excluded from the analysis. The decision of a ZF or conventional fluoroscopic (CF) approach was made by operator's preference, and a transition from ZF to CF was likewise chosen by the operator if deemed necessary. The study was approved by the local Ethical Committee (KEK-ZH-NR: BASEC 2016-00116) and was performed according to the Declaration of Helsinki and guidelines for good clinical practice.

### Data Collection and Analysis

Procedure time was defined from the time of groin puncture until removal of all venous sheaths. We defined acute success as bidirectional block for CTI-dependent atrial flutter, non-inducibility for AVNRT and AT, loss of antegrade and retrograde accessory pathway conduction for WPW syndrome, and cessation of premature ventricular beats for premature ventricular contraction. Major complications were defined as cardiac effusion or tamponade, high-grade atrioventricular block, cardiac decompensation, cardiogenic shock, femoral pseudo-aneurysm, arterial-venous fistula, or inguinal haemorrhage requiring intervention. Minor complications were defined as inguinal haemorrhage without intervention, cardiac pain without effusion, and other symptoms without need of intervention but prolonging hospital stay. Dose area product and effective dose (ED) were used as measures of radiation exposure instead of total fluoroscopy time, and ED (mSv) was adjusted for sex and age, as previously recommended [1]. Follow-up was conducted by assessing all medical chart data up to 3 months after the intervention. Statistical analysis was performed using JMP® (Version 15; SAS Institute Inc., Cary, NC, USA, 1989–2019). Normality of distribution was assessed using either a Kolmogorov-Smirnov or Shapiro-Wilk test. Continuous variables are expressed as mean ± standard deviation, while categorical variables are expressed as absolute numbers and percentages. Comparison between variables was performed using a χ^2^ test, Student's *t* test, or Mann-Whitney U test, as appropriate. *p* values <0.05 were considered statistically significant.

### Procedure Details

In January 2019, we implemented a structured and standardized workflow for a ZF approach for diagnosis and treatment of right-sided cardiac arrhythmias using the CARTO3 navigation system (Carto3; Biosense Webster, Diamond Bar, CA, USA). All interventions were performed by the same three experienced electrophysiologists, and ZF or CF approach was selected by their preference. If ZF approach was chosen, fluoroscopy was always available as a “bail-out” strategy. All interventions were performed with local anaesthesia and conscious sedation. For AVNRT, a non-irrigated ablation catheter without contact force sensor was used, while for CTI ablation, an irrigated contact sensor-enabled ablation catheter was chosen. For all other procedures, ablation catheters used were based on operator preference.

### ZF Approach

**Fig. 1:**
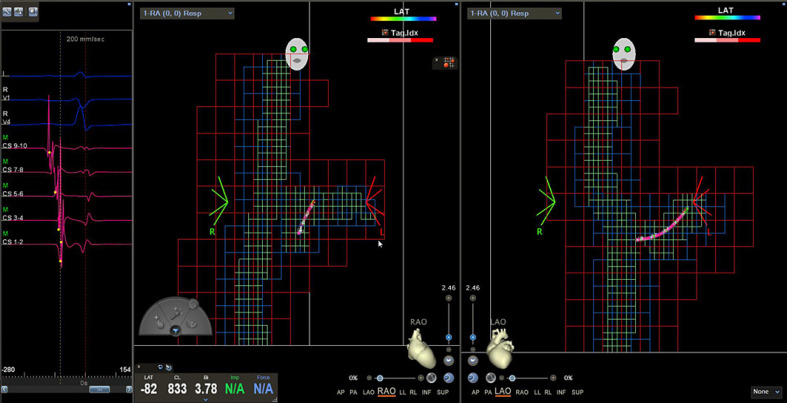
Visualization matrix. Visualization matrix created by the magnetic decapolar catheter (Decanav; Biosense Webster), afterwards placed in the CS. Left: electrical signals from corresponding electrode pairs. Middle: RAO 45°. Right: LAO 45°. CS, coronary sinus; RAO, right anterior oblique; LAO, left anterior oblique.

**Fig. 2:**
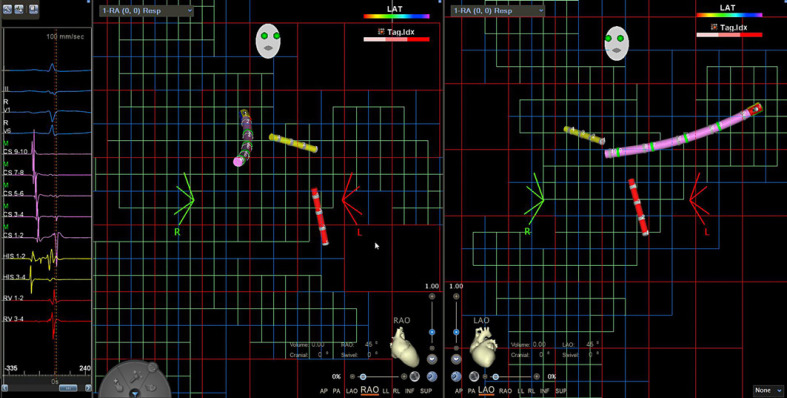
Diagnostic catheters in position. Left: electrical signals from corresponding catheters. Middle: RAO 45° view. Right: LAO 45° view. Pink: decapolar catheter in the CS. Red: quadripolar catheter in right ventricle. Yellow: quadripolar catheter in His position. CS, coronary sinus; RAO, right anterior oblique; LAO, left anterior oblique.

After femoral venous puncture and sheath insertion, a steerable decapolar diagnostic catheter with a magnetic sensor (Biosense Webster Decanav^TM^, Biosense Webster®) was advanced to the right atrium using the CARTO3 mapping system for visualization. Due to the magnetic sensor, the catheter tip is visualized from the time of vascular entrance. The occurrence of electrical signals displayed from the catheter's electrodes coinciding with P-waves on the surface electrocardiogram confirmed successful advancement into the right atrium. Using standard views (RAO 45°, LAO 45°), the catheter was used to create a visualization matrix for non-magnetic diagnostic catheters to be visualized within the 3D mapping system (Fig. 1; online suppl. Video 1; for all online suppl. material, see www.karger.com/doi/10.1159/000526564) and to achieve a rough anatomic overview of the right-sided heart chambers. To achieve this, the catheter was advanced into the superior and inferior vena cava, the area around the interatrial septum as well as the tricuspid annulus and the right ventricle before being placed in the coronary sinus (CS). Since FamDX^TM^ (Biosense Webster®) was not yet available during the procedures of this study, anatomical reconstruction was not feasible without connected ablation catheters, so the visualization matrix was the only guidance. Two non-magnetic quadripolar catheters were advanced into the right atrium from the groin. As mentioned above, these were only able to be visualized once they reached the area of the visualization matrix, which usually corresponded to the level just below the liver veins. Before that, advancement was guided by tactile feedback. Once visualized, placement in the right ventricle as well as in the region of the His bundle was achieved using a combination of anatomic orientation by the navigation system, interpretation of electrical signals, and characteristic catheter movement (Fig. 2; online suppl. Video 2).

**Fig. 3:**
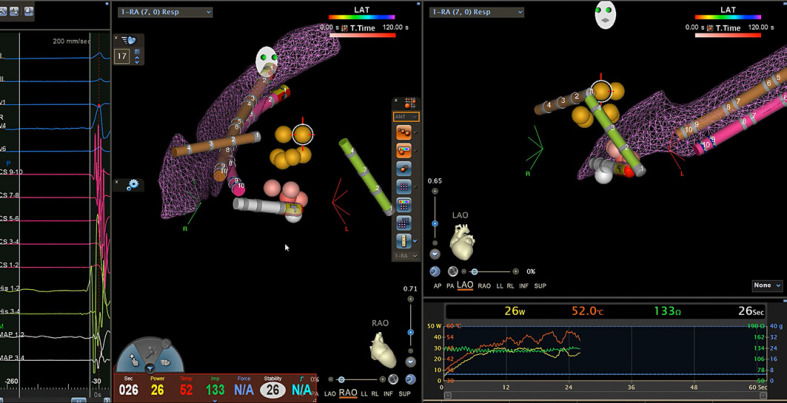
Slow pathway ablation. Left: electrical signals of a junctional beat during ablation. Middle: RAO 45° view. Right superior: LAO 45° view. Right inferior: graphical distribution of power (Watt), temperature (°C), and impedance (Ohm) during ablation. Orange points: His region. Pink points: ablation tags. White point: annotated slow pathway region. Pink catheter: decapolar catheter within the CS. Green catheter: quadripolar catheter in the right ventricle. Brown catheters: catheter images (“snapshots”) from previous catheter locations in case of catheter displacement. During ablation, the relation of the ablation catheter to the His position as well as the slow pathway region can be observed continuously on the navigation system. AVNRT, atrioventricular-nodal reentry-tachycardia; CS, coronary sinus; RAO, right anterior oblique; LAO, left anterior oblique.

After EPS and determination of induced arrhythmia mechanism, the ablation catheter was advanced to the right atrium from the groin similar to the diagnostic decapolar catheter due to incorporated magnetic sensors, achieving visualization once inserted into the venous blood pool. If long sheaths were used for stabilization, a long J-wire was inserted into the venous system. The long sheath was advanced over the wire for only about 10 cm without fluoroscopic guidance before removing wire and dilator. Then, blood was aspirated from the side-arm of the long sheath (confirming intravenous position) and flushed. The ablation catheter was advanced over the long sheath into the right atrium. Next, the long sheath was advanced over the ablation catheter up to its distal end. Surrounding important structures (CS, His bundle, tricuspid annulus) were marked and visualized in the navigation system by either manual or automatic point collection. Anatomical and activation mapping was performed with either an ablation catheter or a 20-pole diagnostic catheter (Pentaray Nav eco Catheter; Biosense Webster) if necessary. Ablation catheter selection, power settings, and ablation point annotation (manual vs. automatic annotation by the navigation system) were left to the operator's decision. All arrhythmia approaches followed standardized workflows concerning mapping and ablation strategies (Fig. 3).

### Conventional Fluoroscopic Approach

After venous puncture in the groin and sheath insertion, a decapolar diagnostic catheter without a magnetic sensor (Boston Scientific Dynamic XT^TM^, Boston Scientific®, Marlborough, MA, USA) and one or two quadripolar diagnostic catheter were advanced to the right atrium and placed in the CS, at the His bundle, or the right ventricular apex using fluoroscopy for navigation. After the diagnostic EPS, further management depended on the underlying arrhythmia. Long sheaths and ablation catheters were placed in the right atrium under fluoroscopic guidance. However, for all catheter manipulation and ablation procedures, the electroanatomical navigation system was always available to the operator, hence fluoroscopy was used “as needed but as low as reasonably achievable” by the operator.

## Results

### Study Population and Baseline Characteristics

Between January 2019 and December 2020, 412 consecutive patients underwent an EP procedure for right-sided cardiac arrhythmias at our institution. After exclusion of patients with endocardial devices, undergoing focal cryoablation or receiving additional procedures during the same intervention necessitating fluoroscopy, 328 consecutive patients were included in the analysis. Among these 328, 126 patients underwent a CF approach, whereas 202 underwent a ZF approach. Apart from atrial fibrillation being more common in the ZF group (ZF 33 vs. CF 11, *p* = 0.044), other baseline comorbidities such as mean age (54 ± 17.8 vs. 57 ± 18.5, *p* = 0.908) and sex distribution (male 115 vs. 73, *p* = 0.858) did not differ between the groups (Table 1). Interventions were evenly distributed among the two groups except CTI ablation for typical atrial flutter, which was significantly more often performed in the ZF group (57 vs. 18, *p* = 0.003) and WPW ablation, which was significantly more often performed in the CF group (9 vs. 13, *p* = 0.025).

### Procedure Characteristics

**Fig. 4:**
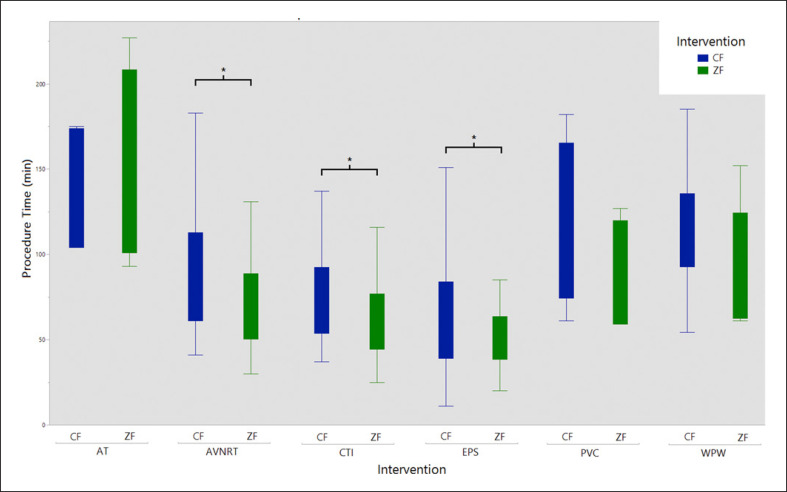
Procedure time per type of cardiac arrhythmia for CF and ZF approach. CF, conventional fluoroscopy; ZF, zero-fluoroscopy; AT, atrial tachycardia; AVNRT, atrioventricular-nodal reentry-tachycardia; EPS, electrophysiological study; PVC, premature ventricular contractions; WPW, Wolff-Parkinson-White syndrome. *Significant difference in statistical analysis.

Mean procedure time (70 ± 36 vs. 87 ± 44 min, *p* = 0.0001) was significantly lower in the ZF group (Table 2), which was driven by the lower mean duration time of diagnostic EP studies (51 ± 20 vs. 71 ± 52 min, *p* = 0.033), AVNRT (72 ± 29 vs. 88 ± 37 min, *p* = 0.003), and CTI interventions (67 ± 40 vs. 77 ± 38 min, *p* = 0.032, Table 2; Fig. 4). Mean ablation time (234 ± 288 vs. 284 ± 323 s, *p* = 0.157) was similar between the two groups. Of 328 cases, 27 were redo cases with previous interventions (10 vs. 17, *p* = 0.878). The mean amount of fluoroscopy used in the CF group was 95 ± 157 μG/m^2^, resulting in a mean ED of 0.2 ± 0.4 mSv for all interventions (Table 3). Highest fluoroscopy doses were used for AT, AVNRT, and EPS (Table 3).

### Complication Rate and Follow-Up

Periprocedural complications occurred in 3% of patients (major complications 1%) without a significant difference between the groups. The one major complication in the ZF group was a cardiac decompensation after CTI ablation that required prolonged hospital stay and intravenous diuretics without further sequelae. Two minor complications in the ZF group consisted of 1 patient with transient pericardial chest pain (without pericardial effusion or alterations on electrocardiography) with spontaneous resolution within days, and 1 patient suffering from a peri-interventional mild allergic reaction. The two major complications in the CF group were a cardiogenic shock after CTI ablation and a pericardial effusion necessitating drainage. The three minor complications in the CF group consisted of 1 patient with transient pericardial chest pain (without pericardial effusion or alterations on electrocardiography), 1 patient with groin haematoma at the venous puncture site necessitating prolonged hospital stay, and 1 patient with a haematoma at the peripheral venous access site. None of the complications left permanent sequelae. During a mean follow-up of 240 ± 108 days, 10 patients suffered from a documented arrhythmia recurrence (0.6% in ZF group vs. 9.7% in the CF group, *p* = 0.0005, Table 4).

## Discussion

In this retrospective cohort study, we demonstrate that a ZF approach using only electroanatomic mapping during catheter interventions for diagnosis and treatment of right-sided cardiac arrhythmias is feasible, efficient, and safe, without increasing complications or duration of the procedure compared to a CF approach. To the best of our knowledge, our report is the largest cohort control study to report on zero-fluoro EP procedures for diagnosis and treatment of right-sided cardiac arrhythmias using only electroanatomic mapping without additional invasive procedures.

### Feasibility, Efficiency, and Safety

Acute success during the procedure did not significantly differ between the groups (CF 97.9 vs. ZF 100%, *p* = 0.399), while procedure time was lower in the ZF group (CF 87 ± 44 vs. ZF 70 ± 36 min, *p* = 0.0001), suggesting adequate feasibility and efficiency of the ZF approach. We did not observe increased complications in the ZF group compared to the CF group (CF 4 vs. ZF 2%, *p* = 0.292), and the observed complications did not seem to be related specifically to the ZF approach, suggesting adequate safety of a ZF approach. During follow-up, a statistically higher number of documented relapse of arrhythmia was observed in the CF group (CF 9.7% vs. ZF 0.6%). The observed differences in relapse and procedure time may primarily be explained by retrospective bias due to transitioning of difficult interventions from a ZF over to a CF approach, as fluoroscopy was always available as bail-out, limiting the statistical value of the comparison. Additionally, a higher proportional amount of WPW was performed in the CF group (10 vs. 4% of all cases, *p* = 0.025, Table 1), maybe because of safety concerns due to atrioventricular block or difficulties in sheath maneuveribility with the ZF approach but potentially increasing complications and decreasing long-term success of the CF group. However, our primary aim was to demonstrate the feasibility, efficiency, and safety of a ZF approach, not demonstrating shorter procedure times or increased efficacy compared to a CF approach. Additionally, the low overall amount of documented relapses in the ZF group during follow-up (0.5%) also suggests a sufficient efficacy of a primary ZF approach.

These results are in line with several studies evaluating the feasibility, efficiency, and safety of ZF EP procedures for right-sided cardiac arrhythmias in recent years [5, 6, 7, 8, 9, 10, 11, 12, 13, 14, 15, 16, 17, 18, 19]. However, previous trials have evaluated ZF catheter interventions during EP procedures with either a considerably lower amount of patients examined [5, 7, 8, 12], without a control group within the same time period [5, 6, 10, 14, 16], without complete elimination of fluoroscopy [11, 18], exclusively in combination with ICE [6, 14], after previous trans-oesophageal EPS [13], with primary use of ablation catheters [9, 14, 17, 19] or after excluding diagnostic EPS [5, 6, 8, 9, 10, 12, 13, 14, 16, 19]. In this study, ICE was not used in the ZF approach, since besides requiring considerable expertise and the additional cost of ICE, there is a potential of increased morbidity due to an additional venous access and an indwelling catheter. Similarly, a primary use of ablation catheters does not seem economically effective, and excluding diagnostic EPS does not reflect general clinical practice and hence limits the informative value concerning efficiency and safety of ZF catheter interventions for EP procedures. Trans-oesophageal EPS is not routinely performed at our centre, but the additional invasiveness may also limit the implementation in daily practice. Our study is the first to show that, without the above limitations, a primary ZF approach with electroanatomic mapping only is feasible, efficient, and safe for diagnosis and treatment of right-sided cardiac arrhythmias, without increasing procedure time or complications compared to a primary CF approach.

### Fluoroscopy versus Electroanatomic Mapping

We reached radiation ED <1 mSv for all our procedures (Table 3), which is expected to have a minimal long-term deterministic radiation risk for our patients [4]. The most important principles to reduce fluoroscopy exposure are physician attitude towards fluoroscopy use (As Low As Reasonably Achievable [ALARA]) and general measurements (i.e., reduced frame rate, tube-to-intensifier distance, lead glasses, etc.) [1]. The advent of non-fluoroscopic three-dimensional electroanatomical mapping systems in the late 1990s reduced the amount of fluoroscopy needed for EP procedures substantially, and randomized studies proved similar efficacy and safety of electroanatomical mapping for navigation compared to continuous fluoroscopy [20]. Our data indicate that the full potential of today's electroanatomic mapping systems will ultimately obliviate the need of fluoroscopy for the treatment of right-sided cardiac arrhythmias in a substantial amount of cases. While the ZF approach exhibits equal safety and efficiency compared to the CF approach using ALARA, the potential downside represents increased costs due to the electroanatomic mapping system and the magnetic sensor-enabled catheters, as well as a considerable learning curve. In our daily practice, the electroanatomic mapping system is always available in our EP laboratory, but the price of a decapolar catheter with a magnetic sensor is roughly 6% more than the price of a decapolar catheter without a magnetic sensor − however, the price difference may vary on an institutional basis. On the other hand, the potential elimination of the detrimental long-term effects of fluoroscopy represents a major benefit and may render price differences worthwhile: reducing fluoroscopy by electroanatomical mapping has been shown to permit a 96% reduction in estimated risk of cancer incidence and mortality of patients, as well as a reduction in estimated years of life lost and affected [11]. Accordingly, we suggest the primary ZF approach for diagnosis and treatment of right-sided cardiac arrhythmias as the next potential evolution of the “ALARA” principle introduced more than a decade ago, with backup fluoroscopy whenever medically needed.

## Conclusion

For diagnosis and treatment of right-sided cardiac arrhythmias, a primary ZF approach is feasible, efficient, and safe, without increasing procedure time or complications compared to a primary CF approach. However, fluoroscopy should always be readily available as a bail-out strategy for difficult procedures.

### Limitations

The main limitation of our study is the non-randomized retrospective design, allowing a potential selection bias, prohibiting a comprehensive analysis of the failure rate of our ZF approach, and limiting the outcome analysis concerning recurrence of arrhythmia. Because of the retrospective design, transitioning of patients from ZF to CF was not routinely evaluated in this study. However, based on a very low radiation exposure of some patients in the CF group (ED <0.01), we can assume that at least 14 patients transitioned from a ZF to a CF approach, resulting in a potential “failure rate” of the ZF approach of 6.5%. This underlines the importance of always having fluoroscopy available as a bail-out strategy. However, a large amount of procedures (*n* = 202, 93.5% of all ZF procedures if accounting for the 14 patients that potentially transitioned to the CF group) were successfully performed with the ZF approach with a low recurrence rate (*n* = 1), suggesting feasibility of this approach for most patients. Prospective randomized studies evaluating the failure rate and outcome without the associated retrospective bias and confounding are needed for further insight into ZF catheter interventions in EP procedures.

## Statement of Ethics

This study protocol was reviewed and approved by the local Ethical Commission of Zurich, approval number BASEC 2016-00116. Written informed consent and consent for publication was acquired. This study was conducted according to the guidelines for good clinical practice and the Declaration of Helsinki.

## Conflict of Interest Statement

Dr. Hofer reports educational grants, consultant or speaker fees, and fellowship support from Abbott, Medtronic, Biotronik, Boston Scientific, Biosense Webster, Novartis, Bayer, Pfizer, and Spectranetics/Philips. Dr. Breitenstein has received consultant and/or speaker fees from Abbott, Bayer Healthcare, Biosense Webster, Biotronik, Boston Scientific, Bristol-Myers Squibb, Cook Medical, Daiichi Sankyo, Medtronic, Pfizer, and Spectranetics/Philips. Prof. Steffel has received consultant and/or speaker fees from Abbott, Amgen, Astra-Zeneca, Bayer, Berlin-Chemie, Biosense Webster, Biotronik, Boehringer-Ingelheim, Boston Scientific, Bristol-Myers Squibb, Daiichi Sankyo, Medscape, Medtronic, Merck/MSD, Novartis, Portola, Roche Diagnostics, Pfizer, Portola, Saja, Servier, and WebMD. He reports ownership of CorXL. Prof. Steffel has received grant support through his institution from Abbott, Bayer Healthcare, Biosense Webster, Biotronik, Boston Scientific, Daiichi Sankyo, and Medtronic. Prof. Duru reports educational grant support to his institution from Abbott, Biotronik, Biosense Webster, Boston Scientific, and Medtronic. Dr. Saguner has received educational grants through his institution from Abbott, Biotronik, Biosense Webster, Boston Scientific, and Medtronic. He has received lecture honoraria from Bayer Healthcare, BMS-Pfizer, and Daiichi-Sankyo. He reports stock of Gilead Sciences. Dr. Graup reports stock of Bayer Pharmaceuticals and Novartis.

## Funding Sources

No funding has been received in context to this study.

## Author Contributions

Dr. D. Hofer, Prof. J. Steffel, Prof. F. Duru, Dr. A. Saguner, and Dr. A. Breitenstein contributed to initiation of the study, recruiting and treating patients with a ZF or CF approach, collecting patient data, and writing and critically correcting the manuscript. Dr. T. Sasse and Dr. V. Graup contributed by collecting patient data, conducting the statistical analysis, and writing and critically correcting the manuscript.

## Data Availability Statement

All data generated or analysed during this study are included in this article. Further enquiries can be directed to the corresponding author upon urgent request and associated need, while our upmost intention is to protect our patient's privacy.

## Supplementary Material

Video 1 Supplemental Video

Video 2 Supplemental Video

## Figures and Tables

**Table 1:** Study population

	All (*n* = 328)	CF (*n* = 126)	ZF (*n* = 202)	*p* value
Age	55±18.1	57±18.5	54±17.8	0.908
Male, *n* (%)	188 (57)	73 (58)	115 (57)	0.858
LVEF <50%, *n* (%)	47 (14)	18 (14)	29 (14)	0.986
Coronary artery disease, *n* (%)	33 (10)	12 (10)	21 (10)	0.798
Atrial fibrillation,* *n* (%)	44 (13)	11 (9)	33 (16)	0.044
Oral anticoagulation, *n* (%)	88 (27)	25 (20)	63 (31)	0.870
AT, *n* (%)	9 (3)	4 (3)	5 (2)	0.708
AVNRT, *n* (%)	138 (42)	54 (43)	84 (42)	0.820
CTI, *n* (%)	75 (23)	18 (14)	57 (28)	0.003
EPS, *n* (%)	73 (22)	33 (24)	40 (20)	0.179
PVC, *n* (%)	11 (3)	4 (3)	7 (3)	0.887
WPW, *n* (%)	22 (7)	13 (10)	9 (4)	0.025
Redo, *n* (%)	27 (8)	10 (8)	17 (8)	0.878

CF, conventional fluoroscopy; ZF, zero-fluoroscopy; LVEF, left ventricular ejection fraction; AT, atrial tachycardia; AVNRT, AV-nodal reentry-tachycardia; CTI, cavotricuspid isthmus ablation; EPS, electrophysiologic study; PVC, premature ventricular contraction ablation; WPW, Wolff-Parkinson-White ablation.

* Co-presence of atrial fibrillation, which was not ablated.

**Table 2:** Procedure characteristics

	All (*n* = 328)	CF (*n* = 126)	ZF (*n* = 202)	*p* value
Procedure time, min	77±40	87±44	70±36	0.0001
AT (*n* = 9, CF = 4, ZF = 5)	151±49	138±40	161±57	0.529
AVNRT (*n* = 138, CF = 54, ZF = 84)	79±33	88±37	72±29	0.003
CTI (*n* = 75, CF = 18, ZF = 57)	69±39	77±38	67±40	0.032
EPS (*n* = 73, CF = 33, ZF = 40)	60±39	71±52	51±20	0.033
PVC (*n* = 11, CF = 4, ZF = 7)	101±38	118±50	91±29	0.271
WPW (*n* = 22, CF = 13, ZF = 9)	107±37	115±38	96±34	0.229
Fluoro DAP, µG/m^2^		95±157	0	
Fluoro ED, mSv		0.2±0.4	0	
Ablation time, s	264±311	234±288	284±323	0.157
Acute success, *n* (%)	255*	91* (97.9)	162* (100)	0.399

CF, conventional fluoroscopy; ZF, zero-fluoroscopy; AT, atrial tachycardia; AVNRT, AV-nodal reentry-tachycardia; CTI, cavotricuspid isthmus ablation; EPS, electrophysiologic study; PVC, premature ventricular contraction ablation; WPW, Wolff-Parkinson-White ablation; DAP, dose area product; ED, effective dose.

* Considering that EPS procedures in both groups were excluded for this calculation.

**Table 3:** Radiation exposure by intervention

	Fluoro DAP, µG/m^2^	Fluoro ED, mSv
All (*n* = 126)	95±157	0.2±0.4
AT	114±202	0.2±0.4
AVNRT	109±189	0.3±0.5
EPS	110±163	0.3±0.4
WPW	62±85	0.1±0.1
CTI	53±54	0.1±0.1
PVC	46±81	0.09±0.1

AT, atrial tachycardia; AVNRT, AV-nodal reentry-tachycardia; EPS, electrophysiologic study; WPW, Wolff-Parkinson-White ablation; CTI, cavotricuspid isthmus ablation; PVC, premature ventricular contraction ablation; DAP, dose area product.

**Table 4:** Follow-up

	All	CF	ZF	
	(*n* = 328)	(*n* = 126)	(*n* = 202)	*P* value
Complications, *n* (%)	9 (3)	5 (4)	4 (2)	0.292
Minor complications, *n* (%)	6 (1.8)	3 (2.3)	3 (1.4)	0.561
Major complications, *n* (%)	3 (1)	2 (1.5)	1 (0.5)	0.321
Documented relapse, *n* (%)	10 (3.9*)	9 (9.7*)	1 (0.6*)	0.001
AT	2	1	1	
AVNRT	0	0	0	
WPW	2	2	0	
CTI	3	3	0	
PVC	3	3	0	

AT, atrial tachycardia; AVNRT, AV-nodal reentry-tachycardia; WPW, Wolff-Parkinson-White ablation; CTI, cavotricuspid isthmus ablation; PVC, premature ventricular contraction ablation.

* Considering that EPS procedures in both groups were excluded for this calculation.
